# Factors influencing wind turbine avoidance behaviour of a migrating soaring bird

**DOI:** 10.1038/s41598-022-10295-9

**Published:** 2022-04-19

**Authors:** Carlos D. Santos, Hariprasath Ramesh, Rafael Ferraz, Aldina M. A. Franco, Martin Wikelski

**Affiliations:** 1 Department of Migration, Max Planck Institute of Animal Behavior, Am Obstberg 1, 78315 Radolfzell, Germany; 2 Núcleo de Teoria e Pesquisa do Comportamento, Universidade Federal do Pará, Rua Augusto Correa 01, Guamá, Belém, 66075-110 Brazil; 3 CESAM-Centro de Estudos do Ambiente e do Mar, Faculdade de Ciências, Universidade de Lisboa, Campo Grande, 1749-016 Lisbon, Portugal; 4 School of Environmental Sciences, University of East Anglia, Norwich, NR4 7TJ UK; 5 Centre for the Advanced Study of Collective Behaviour, University of Konstanz, 78457 Konstanz, Germany

**Keywords:** Ecology, Conservation biology

## Abstract

Wind energy production has expanded as an alternative to carbon emitting fossil fuels, but is causing impacts on wildlife that need to be addressed. Soaring birds show concerning rates of collision with turbine rotor blades and losses of critical habitat. However, how these birds interact with wind turbines is poorly understood. We analyzed high-frequency GPS tracking data of 126 black kites (*Milvus migrans*) moving near wind turbines to identify behavioural mechanisms of turbine avoidance and their interaction with environmental variables. Birds flying within 1000 m from turbines and below the height of rotor blades were less likely to be oriented towards turbines than expected by chance, this pattern being more striking at distances less than 750 m. Within the range of 750 m, birds showed stronger avoidance when pushed by the wind in the direction of the turbines. Birds flying above the turbines did not change flight directions with turbine proximity. Sex and age of birds, uplift conditions and turbine height, showed no effect on flight directions although these factors have been pointed as important drivers of turbine collision by soaring birds. Our findings suggest that migrating black kites recognize the presence of wind turbines and behave in a way to avoid then. This may explain why this species presents lower collision rates with wind turbines than other soaring birds. Future studies should clarify if turbine avoidance behaviour is common to other soaring birds, particularly those that are facing high fatality rates due to collision.

## Introduction

Wind energy generation has been increasing over the past two decades with the global commitment to transition to renewable energy from carbon-emitting fossil fuels. Wind farms occupying large areas have been sprouting both on land and offshore^1^. With an increase in wind farms around and close to wildlife habitats, reports of impacts on wildlife emerged and have been well documented in the literature^2,3^. Rising bird mortality from direct collision with the blades of functioning turbines has become a burning issue questioning the apparent environmental friendliness of wind energy. While it is important to mitigate climate change, the switch to renewable energy should be made responsibly with minimal impacts to wildlife.

Wind farms can impact birds either directly by collision with turbines or associated structures, or indirectly by displacement, resource exclusion, habitat modification and increased energy expenditures by being a barrier to their regular flight paths^2,4^. Terrestrial soaring birds (most raptors and other broad-winged large birds) are especially susceptible to mortality from collision with turbines because they frequently fly at the height of the rotors swept zone, show reduced flight maneuverability and are attracted to slopes of windy regions that are also favourable to wind power production^2,5^. However, the large variation of collision rates found between species and sites^3,6^ suggests complex behavioural responses of soaring birds to wind turbines that are important to understand.

Birds generally avoid getting close to wind turbines, although the distance at which they tolerate the presence of turbines varies between species and spatiotemporal contexts^7^. Recent studies on soaring birds have established the presence of clearly defined thresholds to approaching wind turbines^8–13^. However, it remains unclear how these thresholds are established, and which variables trigger further approaches that may lead to collisions. We investigated these questions using the black kite (*Milvus migrans*) as a model species for soaring birds. This species is one of the most abundant soaring birds in Western Europe and is particularly susceptible to interact with wind turbines during their post-breeding migration in the region of the Strait of Gibraltar. Here, large numbers of individuals concentrate in areas with high densities of wind turbines^10^. Our analyses were based on a published dataset^14^ of high resolution GPS tracking data recorded for 139 individual birds. The dataset includes GPS positions, altitude, speed and flight heading with a frequency between one second and 20 min, and also discriminates sex and age of each bird tracked. This information was combined with the locations of wind turbines and their characteristics, as well as the wind conditions during the periods of bird tracking, allowing us to address the following specific questions: (1) How does wind turbine proximity affect bird flight directions at different flight heights? (2) Are flight directions near wind turbines influenced by wind direction and speed, uplift, turbine height and bird sex and age? We predict that: (1) birds will be less frequently oriented towards turbines as they fly closer and at low altitude (shown before for other raptor species^8,12^); (2) bird directions will not be affected by turbine proximity when they fly higher than the rotor blades (shown before for other raptor species^12,15^); (3) birds will be oriented more often towards turbines in areas rich in uplift, as they may compromise on avoidance to optimize the use of uplift^5,16^; (4) birds will be less frequently oriented towards turbines when the wind is blowing in that direction in order to avoid being dragged into the rotor swept zone (this hypothesis has not yet been tested or suggested); (5) birds will be less frequently oriented towards larger than smaller turbines as the former have greater visual impact, although earlier studies were unable to prove this hypothesis^2^; (6) sex and age will not influence flight directions, as similar turbine displacement patterns were found in different sexes and age classes of this species^13^.

## Methods

### Data collection

**Figure 1:**
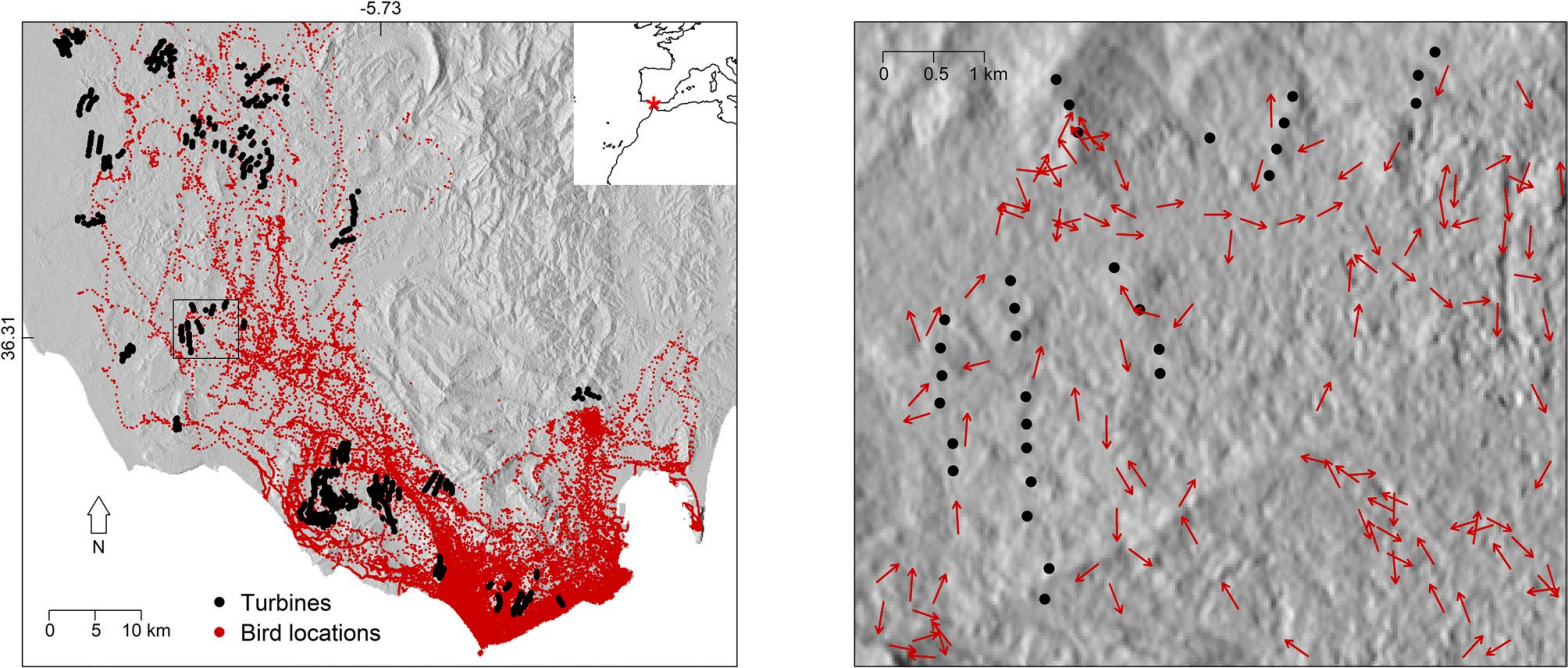
Left panel shows the spatial distribution of bird and turbine locations in the study area between Cadiz and Tarifa (southern Spain). Red asterisk in the top right inset marks the location of the study area. Right panel shows bird flight headings in comparison to turbine locations in a small section of the study area (square in the left panel). Hill shading was added as a background to illustrate the interaction between bird space use and topography. The data used to illustrate hill shading was retrieved from a publicly available digital elevation model (https://lpdaac.usgs.gov).

We used a published dataset^14^ recorded from the GPS tracking of 139 black kites while moving in the region between Cadiz and Tarifa (southern Spain, Fig. 1) during the post-breeding migration seasons in 2012 and 2013. The dataset included GPS locations, altitude, instant speed and heading with frequencies between one second and 20 min, comprising 231, 193 observations, and also included the age and sex of the tracked birds. GPS fixes were recorded every 20 min in the early and late hours of the day (7:30 to 9:30 and 18:30 to 20:30), when birds were less likely to fly, and each minute for the remaining daylight hours. Higher-resolution data (fixes every 10 s with 20-s bursts at 1 Hz every 3 min) were recorded when birds approached the edge of the Strait of Gibraltar at any time of the day. Locations obtained when birds were not flying (speed < 1 m/s) were excluded from analyses. Birds were tagged in Tarifa during periods of high speed crosswinds at the Strait of Gibraltar that restrict their passage to Africa^17,18^ and force them to roam in areas with high density of wind turbines (Fig. 1). Further details on field procedures and tracking equipment can be found in earlier publications with subsets of these data^10,13,19^.

Bird locations were annotated for wind conditions and the characteristics of the closest wind turbine. Wind data were recorded every 10 min at a weather station located in Tarifa (36.0138°N, 5.5988°W), except during two periods (12–13 August 2012 and 6–11 September 2013) for which we used data from a weather station located in San Roque (36.2730°N, 5.2844°W). Both stations belong to the Spanish state meteorological agency (AEMET). Information on the location of wind farms and the number and characteristics of wind turbines (model, rotor diameter and hub height) was obtained from a public database (https://www.thewindpower.net), and we used high resolution aerial photography (available at http://www.juntadeandalucia.es) to locate each turbine within each wind farm. Hub height was not available for some turbine models but for the remaining, hub height was similar in length to rotor diameter. Thus, we assumed that turbine height was 1.5 times the rotor diameter. We also determined uplift conditions at the location of each turbine using the method described in Santos et al.^19^. This method estimates orographic uplift by combining terrain aspect and slope, and wind direction and speed. Terrain aspect and slope were extracted from a digital elevation model (available at https://lpdaac.usgs.gov). Specific values of orographic uplift were calculated for the time when each bird observation was recorded, aiming to account for temporal variations of wind conditions. For thermal uplift, we estimated land surface temperature from two Landsat 8 OLI/TIRS images acquired on 8 and 17 July 2013 (available at http://earthexplorer.usgs.gov), from which uplift velocities were calculated (following Santos et al.^19^). We assumed that variation of thermal uplift in the proximity of different turbines was mostly due to variation of landcover. Temporal variation of uplift at each turbine location should be comparatively low during the summer at the middle hours of the day (10–18 h), when most flight data was collected. We also assumed that thermal uplift derived from images acquired in July 2013 were representative of the post-breeding migration (mid-July to mid-September) in 2012 and 2013. In support of this assumption, Santos et al.^19^ found a high spatial correlation of thermal uplift estimates between different years at this region during the summer.

Both orographic and thermal uplift velocities were calculated for a 500 × 500 m grid in order to obtain a general estimate that included the neighboring areas of each wind turbine. Our estimates did not account for local airflow disturbances caused by the functioning of the turbines, which may influence uplift characteristics in the close proximity of the turbines^20^.

### Data analyses

We modeled flight directions of birds in relation to the location and characteristics of wind turbines, wind conditions, flight height and individual bird traits (sex and age). We were interested in understanding which conditions affected the probability of birds to be oriented towards turbines. Birds were expected to avoid a narrow range of directions that would bring them closer to turbines, but they were not expected to show a preference for any other particular direction. In order to reproduce this expected behaviour, we converted the angular difference between bird heading and the nearest turbine bearing into a binomial variable, where observations were assigned to 1 if the bird’s flight heading deviated less than 60° from the bearing to the nearest turbine or 0 otherwise. The 60° threshold was chosen because we observed an inflated proportion of heading-bearing angular differences between 60° and 90° (Fig. S1), likely reflecting the most common turning angles of birds actively avoiding wind turbines when previously in a collision trajectory. Previous analyses with subsets of this data showed that birds exhibited inflated densities around 700–800 m from the wind turbines^10,13^, probably corresponding to trajectory changes with similar turning angles intended to avoid approaching wind turbines further. This pattern was also observed in other migrating raptors tracked by radar (see Fig. 4 of Cabrera-Cruz and Villegas-Patraca^8^).

Our null hypotheses predicted that birds were not selective regarding their flight directions, thus flight directions should present a random distribution. If this was the case, the relative frequency of directions matching heading-bearing angular differences between − 60° and 60° (corresponding to birds facing turbines) should be near 0.33, while the relative frequency of remaining directions should be near to 0.67.

We modelled the probability of birds to be facing turbines at two spatial scales, (1) at a large scale (up to 1500 m of the wind turbines) in relation to wind turbine distance and flight height, and (2) in the close proximity of wind turbines (up to 750 m) in relation to turbine characteristics, wind conditions and individual bird traits. With approach (1), we intended to circumscribe the range of influence of wind turbines on bird directions. We expected a nonlinear relationship between bird directions and the distance to turbines, similar to that found before for space-use^10,13^. Therefore, we used a binomial Generalized Additive Mixed Model (GAMM) where the probability of facing turbines was a binomial response variable (1 facing turbines and 0 not facing turbines) and distance to the nearest turbine was incorporated as a smooth term interacting with flight height, which was categorized into three classes: Low height (up to the maximum turbine height); Medium height (from the maximum turbine height to the height of two turbines); High height (higher than the upper limit of the medium height class). This model also incorporated bird identity as a random effect to control for the influence of individual specific behaviours in our response variable. The model was fitted with the function gamm4 of the R-package gamm4^21^. The analytical approach (2) focused on flight data recorded within the range of influence of wind turbines, i.e. up to 750 m of wind turbines and lower than the height of turbines. These thresholds were based on the results of the GAMM and the findings of two earlier studies using subsets of these data^10,13^. Here, we fitted a binomial Generalized Linear Mixed Model (GLMM) with the probability of facing turbines included as response variable, bird age and sex, the wind component blowing towards the closest turbine (i.e. the wind vector component along the bearing to the closest turbine), turbine height and the uplift conditions at the turbine locations included as fixed effects, and bird identity included as a random effect. However, as we found a high correlation between turbine height and thermal uplift (Pearson’s correlation = 0.77), we decided to fit two different models, the first keeping thermal uplift as a predictor but excluding turbine height, and the second with the opposite configuration. This allowed us to examine the relevance of these two factors on bird directions without incurring multicollinearity problems in the model (see Table S1). Both models were fitted with the function glmer from the R-package lme4^22^. We examined the temporal and spatial autocorrelation of model residuals for the three models. In all, residuals showed higher correlations at small temporal and spatial scales (Figs. S2, S3, S4). However, when excluding the GPS tracking data collected at 1 Hz, model residuals became relatively unbiased (Figs. S2, S3, S4), allowing us to proceed with no further corrections. We used the function correlog from the ncf R-package^23^ to compute spatial correlations and the function acf from the stats R-package^24^ for temporal correlations. Model fit was evaluated through a tenfold cross-validation for the GAMM. In each iteration, the original dataset was randomly split into a training subset (90% of the data) used to fit a model and a testing subset (10% of the data) against which the model predictions were compared. Model accuracy was calculated as the average and standard deviation of the percentage of correct predictions in the 10 iterations. For the GLMMs, we determined marginal and conditional R^2^ with the function r.squaredGLMM from the MuMIn R-package^25^.

## Results

Our modelling dataset included 13,682 GPS locations recorded from 126 birds after filtering out locations of birds that were not flying, locations that were far away from turbines (beyond 1500 m of the closest turbine) and data collected at 1 Hz (see Fig. S5 for horizontal and vertical distribution of observations). Selected locations were in the vicinity of 472 different wind turbines and distributed through an area of more than 2000 km^2^, with a higher concentration in the southeast region, near Tarifa (Fig. 1).

**Figure 2:**
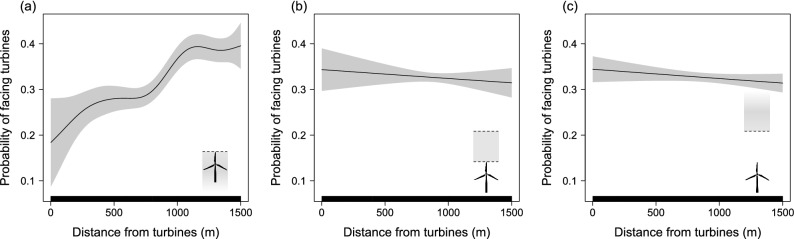
Generalized Additive Mixed Model (GAMM) partial effects of turbine proximity on probability of birds to be oriented towards turbines in three classes of flight height: (a) up to the turbine maximum height; (b) from turbine maximum height to the height of two turbines; (c) higher than the upper limit of class (b). The model response variable was binomial, assigned to 1 if the bird’s flight heading deviated less than 60° from the bearing to the nearest turbine or to 0 otherwise. Bird identity was included as a random effect in the model. Shaded areas represent 95% confidence intervals.

Birds were less likely to be oriented towards turbines as they flew closer to them and at heights lower than the rotor blades (Fig. 2a, Table 1). This effect was non-linear, with the probability of birds to be facing turbines being relatively close to that expected for random flight directions (0.33) at distances farther than 1000 m and dropping quickly between 1000 and 750 m (Fig. 2a). At distances lesser than 750 m, the probability of birds to be facing turbines kept dropping, although the model confidence interval became gradually larger (Fig. 2a) because the number of individuals reaching such close distances to the turbines were very low (Fig. S5). In contrast, there was no effect of turbine proximity on bird directions when they flew higher than the rotor blades, with the probability of birds to be facing turbines matching that expected for random flight directions (0.33, Fig. 2b,c, Table 1).

**Table 1:** Summary statistics of a binomial Generalized Additive Mixed Model (GAMM) relating the probability of birds to be oriented towards wind turbines to their distance to the nearest turbine and flight height. The response variable was assigned to 1 if the bird’s flight heading deviated less than 60° from the bearing to the nearest turbine or 0 otherwise. Flight height was classified into three classes: Low height (up to the maximum turbine height); Medium height (from the maximum turbine height to the height of two turbines); High height (higher than the upper limit of the medium height class). Bird identity was included as a random effect. Model accuracy is represented as the average and standard deviation of the percentage of correct predictions of 10 cross-validation models. *EDF* estimated degrees of freedom, *χ*^*2*^ Chi-square statistic.

Model smooth terms	EDF	*χ*^2^	P-value	Accuracy
s(Distance from turbines): Low height	4.3	64.25	< 0.001	59.2 ± 2.0
s(Distance from turbines): Medium height	1.0	0.56	0.455	
s(Distance from turbines): High height	1.0	1.80	0.180	

**Figure 3:**
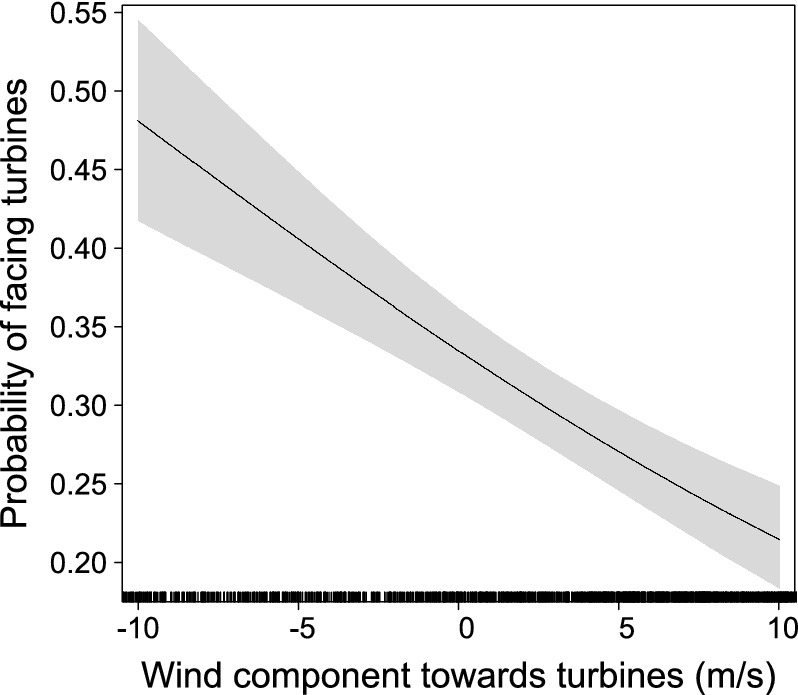
Generalized Linear Mixed Model (GLMM) partial effect of wind component towards turbines on probability of birds to be oriented towards turbines. Partial effect was calculated from the first model of Table 2 (that includes all predictors except turbine height) but the second model delivers identical results (compare model parameters in Table 2). The model response variable was binomial, assigned to 1 if the bird’s flight heading deviated less than 60° from the bearing to the nearest turbine or to 0 otherwise. Bird identity was included as a random effect in the model. Shaded areas represent 95% confidence intervals.

Within the range of 750 m from the wind turbines and up to the height of rotor blades, we found a negative effect of the wind component blowing towards turbines on the probability of birds to be facing turbines, i.e. birds were less likely to be facing turbines when pushed by stronger winds directed to turbines (Fig. 3, Table 2). Turbine height, surrounding uplift, bird age and sex had no significant influence on flight directions.

**Table 2:** Summary statistics of binomial Generalized Linear Mixed Models (GLMMs) relating the probability of birds to be oriented towards wind turbines to individual traits and environmental variables. Because thermal uplift and turbine height were highly correlated (Pearson’s correlation = 0.77) but were both important for the aims of this study, we included each of these variables as predictors in two alternative models. The modelling dataset is restricted to the proximity of turbines (up to 750 m) and low flight height (up to the maximum height of the turbines), where the strongest avoidance is expected (see Fig. 2a). The response variable was assigned to 1 if the bird’s flight heading deviated less than 60° from the bearing to the nearest turbine or 0 otherwise. Bird identity was included as a random effect in both models. Orographic and thermal uplift were estimated only for the turbine locations and did not account with potential airflow disturbance caused by the turbine functioning. Marginal and conditional R^2^ were calculated with the function r.squaredGLMM from the MuMIn R-package^25^. *SE* standard error, *Z* test statistic, *LCI* Lower 95% confidence interval, *UCI* Upper 95% confidence interval.

Model without turbine height	Estimate	SE	Z	LCI	UCI	P	R^2^ cond./marg.
Intercept	− 0.14	1.14	− 0.12	− 2.38	2.13	0.905	0.04/0.03
Age	0.19	0.12	1.59	− 0.05	0.44	0.112	
Sex	− 0.17	0.12	− 1.43	− 0.42	0.06	0.152	
Orographic uplift	0.04	0.14	0.28	− 0.24	0.31	0.779	
Thermal uplift	− 0.30	0.60	− 0.50	− 1.49	0.88	0.617	
Wind comp. towards turbines	− 0.06	0.01	− 6.17	− 0.08	− 0.04	< 0.001	
**Model without thermal uplift**							
Intercept	− 0.84	0.22	− 3.89	− 1.27	− 0.42	< 0.001	0.04/0.03
Age	0.22	0.12	1.82	− 0.02	0.46	0.069	
Sex	− 0.17	0.12	− 1.41	− 0.42	0.06	0.159	
Orographic uplift	0.05	0.14	0.39	− 0.22	0.32	0.697	
Turbine height	0.00	0.00	0.72	0.00	0.01	0.473	
Wind comp. towards turbines	− 0.06	0.01	− 6.14	− 0.08	− 0.04	< 0.001	

## Discussion

Our results show clear wind turbine avoidance behaviour of black kites during migration. The probability of birds to be oriented towards turbines was lower than expected by chance within 1000 m from turbines and dropped considerably at distances less than 750 m (Fig. 2a). These distance thresholds for directional changes match the decline of bird use around wind turbines observed in two earlier studies using subsets of these data^10,13^. These results are also consistent with radar observations of several soaring raptors showing changes of direction at few hundred meters from wind turbines^8^. Importantly, these avoidance patterns were only observed for birds flying up to the height of turbines (Fig. 2). Individuals flying higher did not seem to bother to change their flight directions when getting close to wind turbines, which was also observed earlier for two other raptor species^12,15^. Flight directions of birds entering the area within 750 m of the wind turbines at low height were also affected by the wind component blowing towards the wind turbines. As predicted, birds avoided facing turbines when the wind was pushing them towards turbines (Fig. 3). This suggests that birds recognize the risk of being dragged by the wind to the proximity of the turbines and adjust their flight paths accordingly. This behaviour had not been recorded before for soaring birds.

We found no effects of bird’s age or sex on flight directions despite several studies reporting sex and age differences in turbine collision rates of soaring birds^26–29^. Our results are consistent with those published earlier for this species showing that utilization distribution around wind turbines is not influenced by age or sex^13^. Contrary to our predictions, uplift availability had no effect on flight directions. Previous studies have suggested that soaring birds assume riskier flight behaviours near turbines located in areas rich in uplift^5,16^. Our study area includes several regions with a high orographic uplift, where birds flew dangerously close to wind turbines (see Fig. S6). However, in these regions, birds tended to fly parallel to the rows of turbines, thus avoiding getting close to the rotors swept zone (Fig. S6). The lack of turbine height effect on flight directions was also surprising, as it seems plausible that larger turbines would trigger stronger avoidance responses due to their greater visual impact. Earlier studies with other bird groups also failed to prove this hypothesis^30–32^. However, one study has shown that collision rates in raptors increase with turbine height^33^, which makes our results particularly concerning. Larger turbines have stronger vortices and cause higher air turbulence^20^, which may destabilize the flight of birds from wider distances increasing the risk of collision. This is particularly important as many wind farms are being repowered with larger turbines^34^.

It should be noted that both GLMMs present low marginal and conditional R^2^ (Table 2). This indicates that besides the wind direction, other factors not considered in our models may play a role on the choice of flight directions by the birds near the turbines, such as the level of turbine aggregation, the presence of power lines and other vertical obstacles, or the proximity of feeding and roosting areas. We should also emphasize that turbine avoidance behaviour of soaring birds is not as spatially explicit as in birds that use powered flight^35,36^, as they need to deviate from turbines while optimizing the use of uplift. For this reason, our inference on turbine avoidance behaviour of black kites is probabilistic, based on the poll of flight directions available in the tracking dataset and not on single observations. Consequently, our conclusions accommodate the fact that birds may exhibit flight directions divergent from turbines without being intentionally avoiding them.

Overall, our findings show that black kites respond to the presence of wind turbines, as they adopted avoidance trajectories hundreds of meters far away when flying below the height of turbines, showed a stronger avoidance when pushed by the wind towards the turbines and did not seem to be less responsive at areas with high uplift. Earlier studies on soaring birds showing reduced utilization of the areas around turbines^9,11,12^ and turbine avoidance trajectories^8^ suggest that the perception of turbines is not exclusive of black kites. However, we admit that soaring birds may avoid approaching turbines for reasons other than safety. The functioning of turbines creates air turbulence, particularly in the areas downwind to the turbine rotors^35^, that may compromise uplift generation or destabilize soaring flight. Soaring birds may also avoid turbines due to neophobia, noise or because turbines represent barriers to their movement^2,36^.

The turbine avoidance behaviour of migrating black kites in the study area matches their relatively low collision rate^37^. Other soaring bird species, such as griffon vultures (*Gyps fulvus*) and common kestrels (*Falco tinnunculus*), are much less abundant and present collision rates several fold higher^37,38^. Future studies should investigate if these species and others showing high collision rates with wind turbines present avoidance mechanisms similar to black kites and which factors are attenuating them. This information could support strategies to enhance the perception of wind turbines by soaring birds, such as increasing their visibility^39^ or developing effective deterrents.

## Supplementary Information

Supplementary Information.

## Data Availability

Data available from the Movebank Data Repository 10.5441/001/1.23n2m412.
