# Supplementary material for: Factors influencing wind turbine avoidance behaviour of a migrating soaring bird
Source: Sci Rep. 2022 Apr 19;12:6441. doi: 10.1038/s41598-022-10295-9 (PMC9019107; doi:10.1038/s41598-022-10295-9)
Supplement: Supplementary file 1 — Supplementary Information. [file 41598_2022_10295_MOESM1_ESM.pdf]

Supplementary Information

Factors influencing wind turbine avoidance behaviour of a migrating soaring bird

Carlos D. Santos, Hariprasath Ramesh, Rafael Ferraz, Aldina M. A. Franco, Martin Wikelski

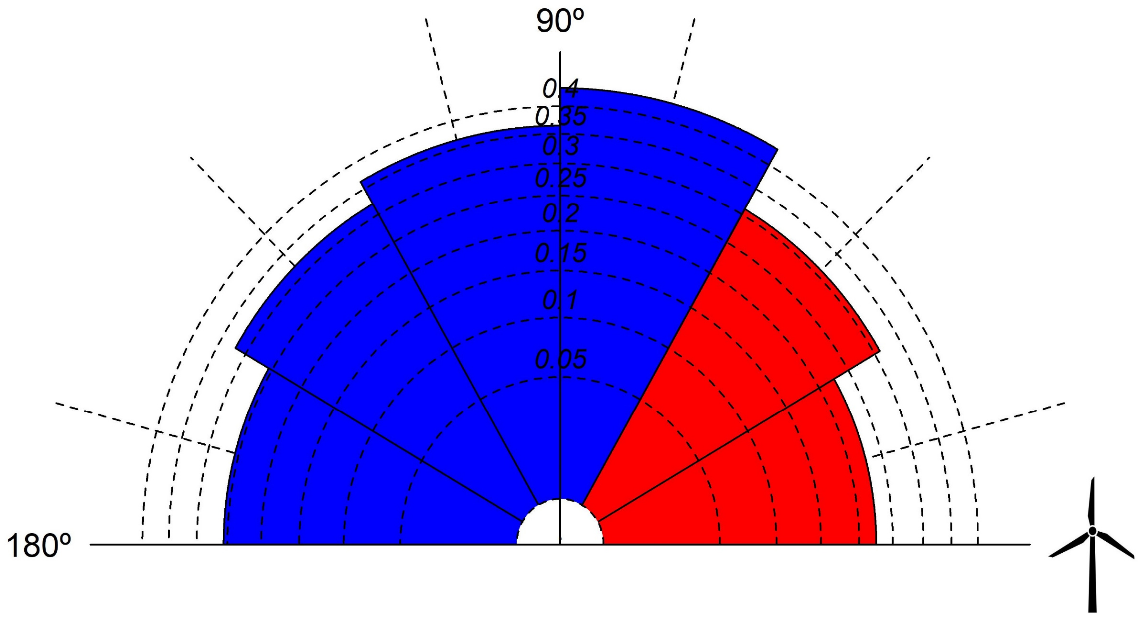

Figure S1. Frequency distribution of angular differences between bird heading at each GPS location and the bearing to the nearest turbine. Angular difference values were rescaled to range between 0 and 180º (i.e. the values between 180 and 360º where subtracted to 360º). Red area covers observations considered to be oriented towards turbines. For this plot we used data up to 800 m of turbines and lower than their height.

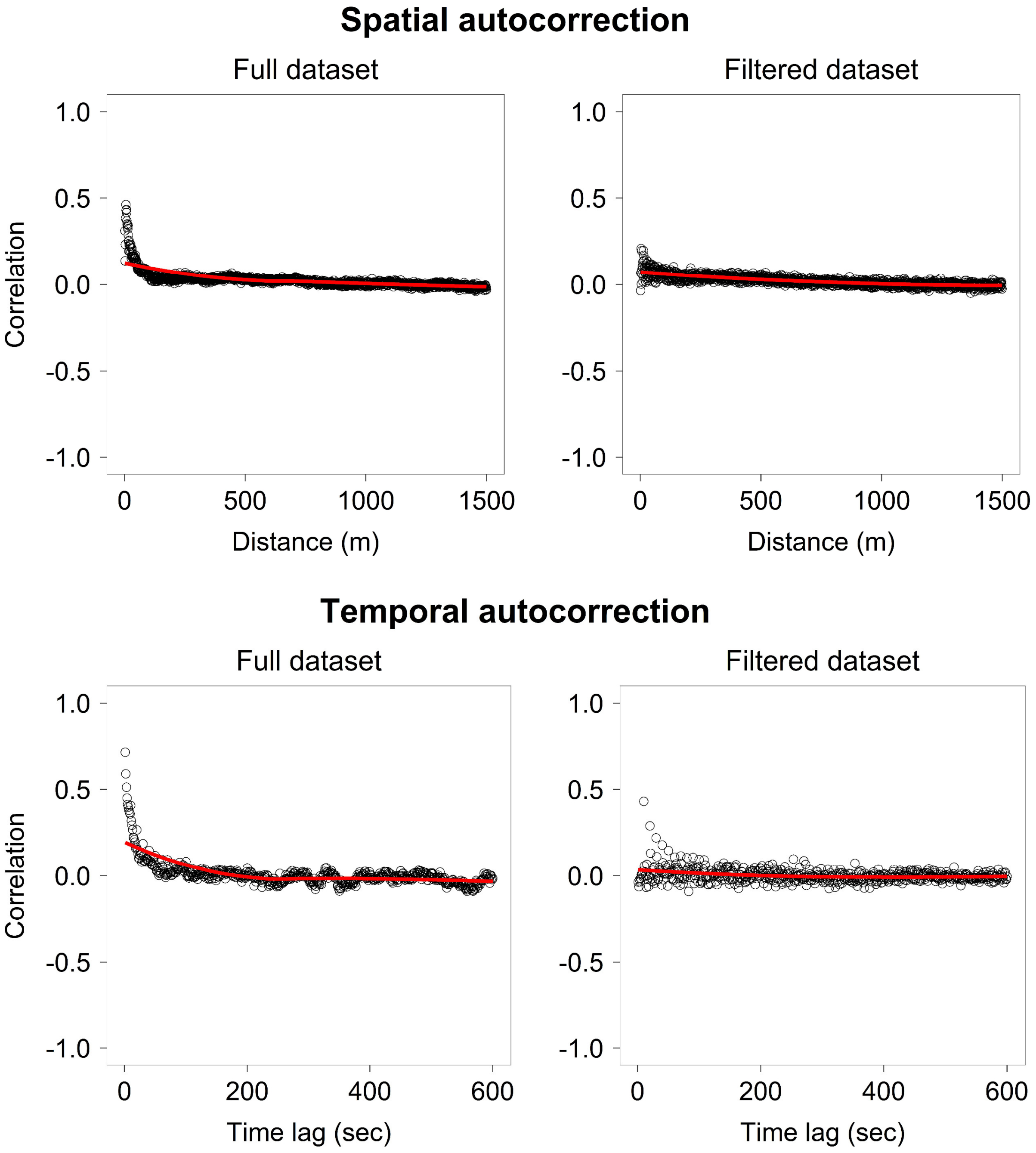

Figure S2. Spatial and temporal autocorrelation of the GAMM residuals. This model relates the probability of birds to be oriented towards turbines to their distance to the nearest turbine and flight altitude. Left and right panels show autocorrelations when the model uses all GPS data available (full dataset) and when it excludes data collected at 1 Hz (filtered dataset) respectively. We used

the function correlog of the ncf R-package[1] to compute spatial correlations and the function acf of the stats R-package[2] for temporal correlations. Trend lines in red are loess curves.

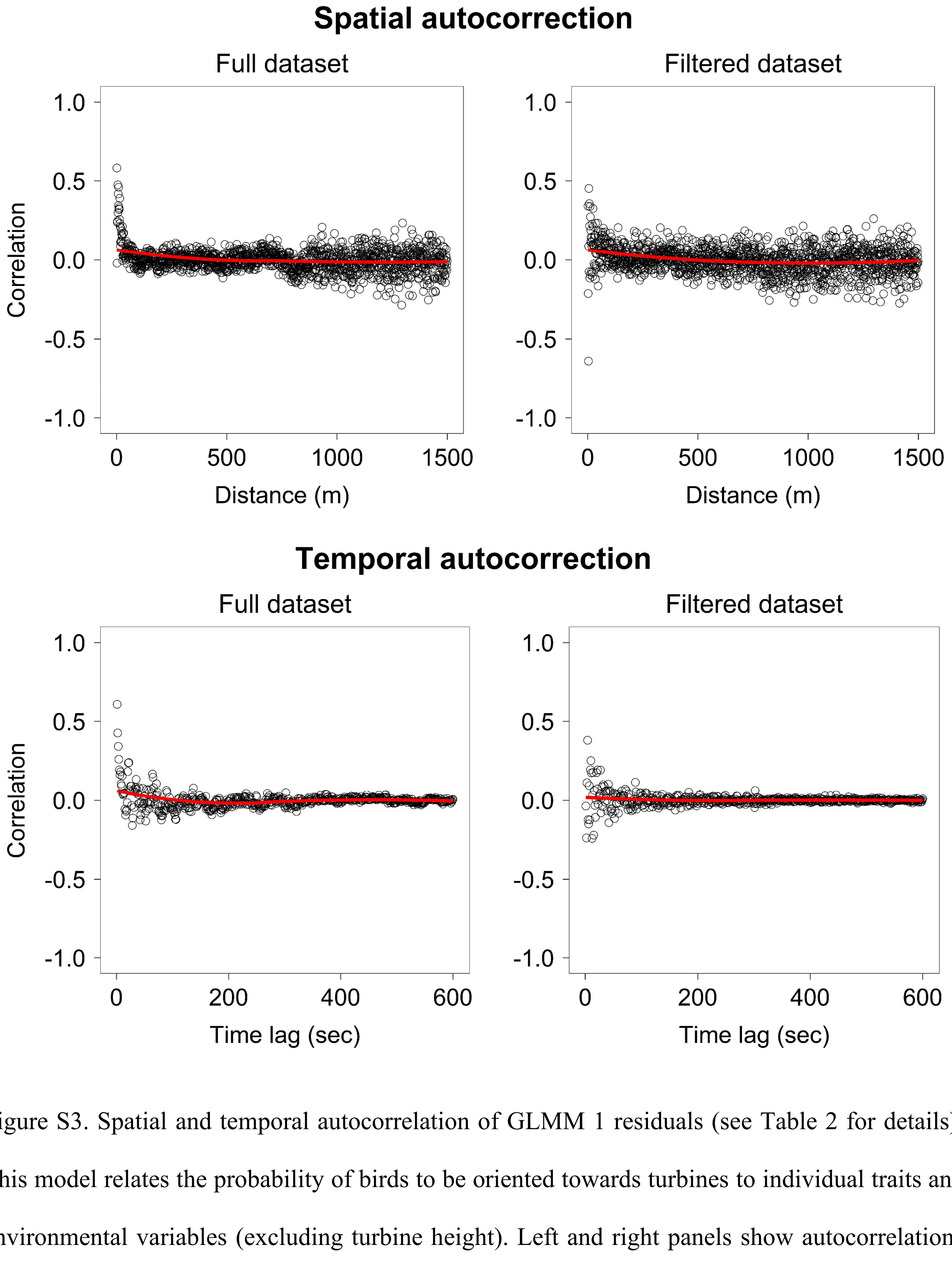

Figure S3. Spatial and temporal autocorrelation of GLMM 1 residuals (see Table 2 for details). This model relates the probability of birds to be oriented towards turbines to individual traits and environmental variables (excluding turbine height). Left and right panels show autocorrelations when the model uses all GPS data available (full dataset) and when it excludes data collected at 1

Hz (filtered dataset) respectively. We used the function correlog of the ncf R-package[1] to compute spatial correlations and the function acf of the stats R-package[2] for temporal correlations. Trend lines in red are loess curves.

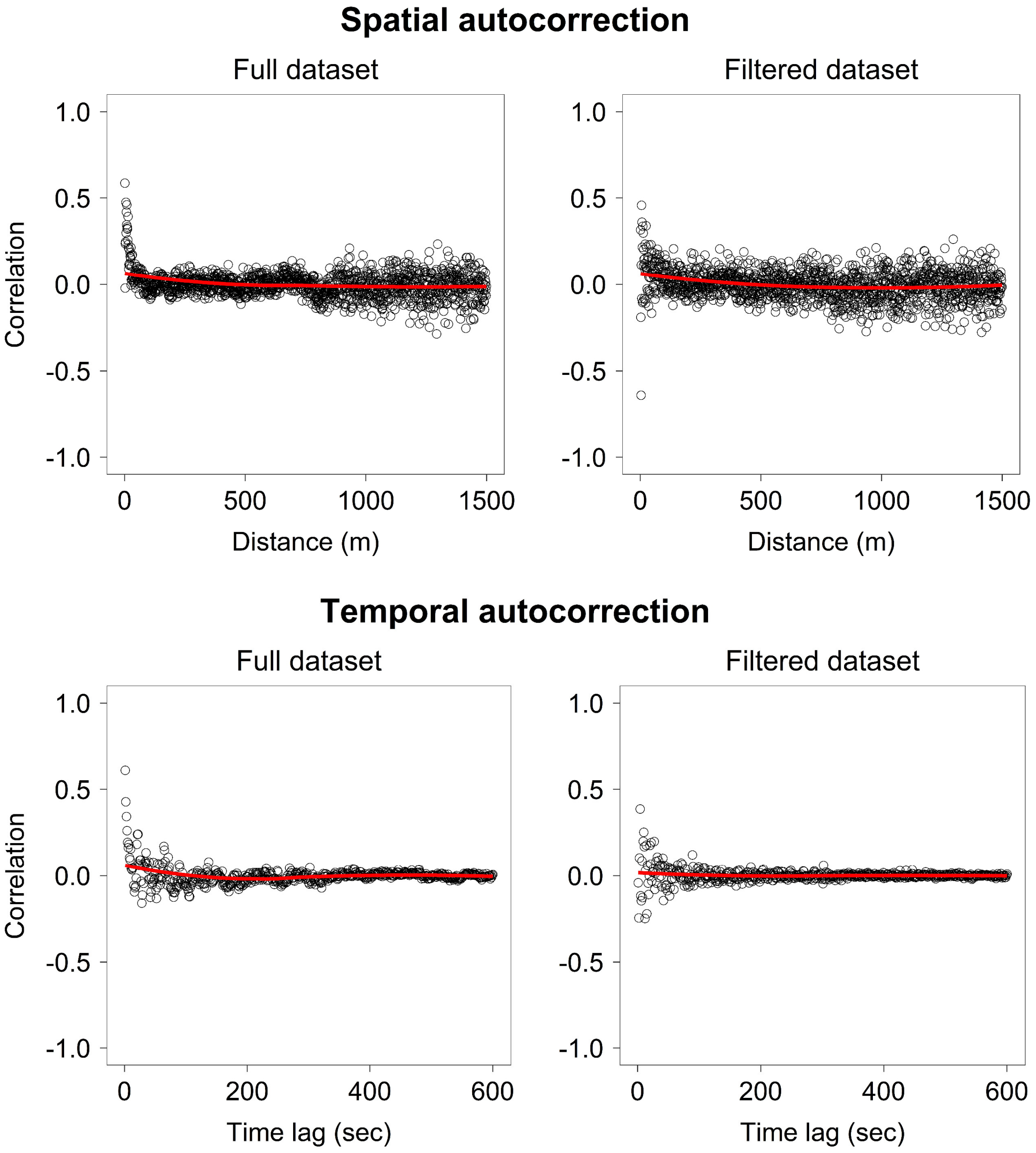

Figure S4. Spatial and temporal autocorrelation of GLMM 2 residuals (see Table 2 for details). This model relates the probability of birds to be oriented towards turbines to individual traits and environmental variables (excluding thermal uplift). Left and right panels show autocorrelations when the model uses all GPS data available (full dataset) and when it excludes data collected at 1

Hz (filtered dataset) respectively. We used the function correlog of the ncf R-package[1] to compute spatial correlations and the function acf of the stats R-package[2] for temporal correlations. Trend lines in red are loess curves.

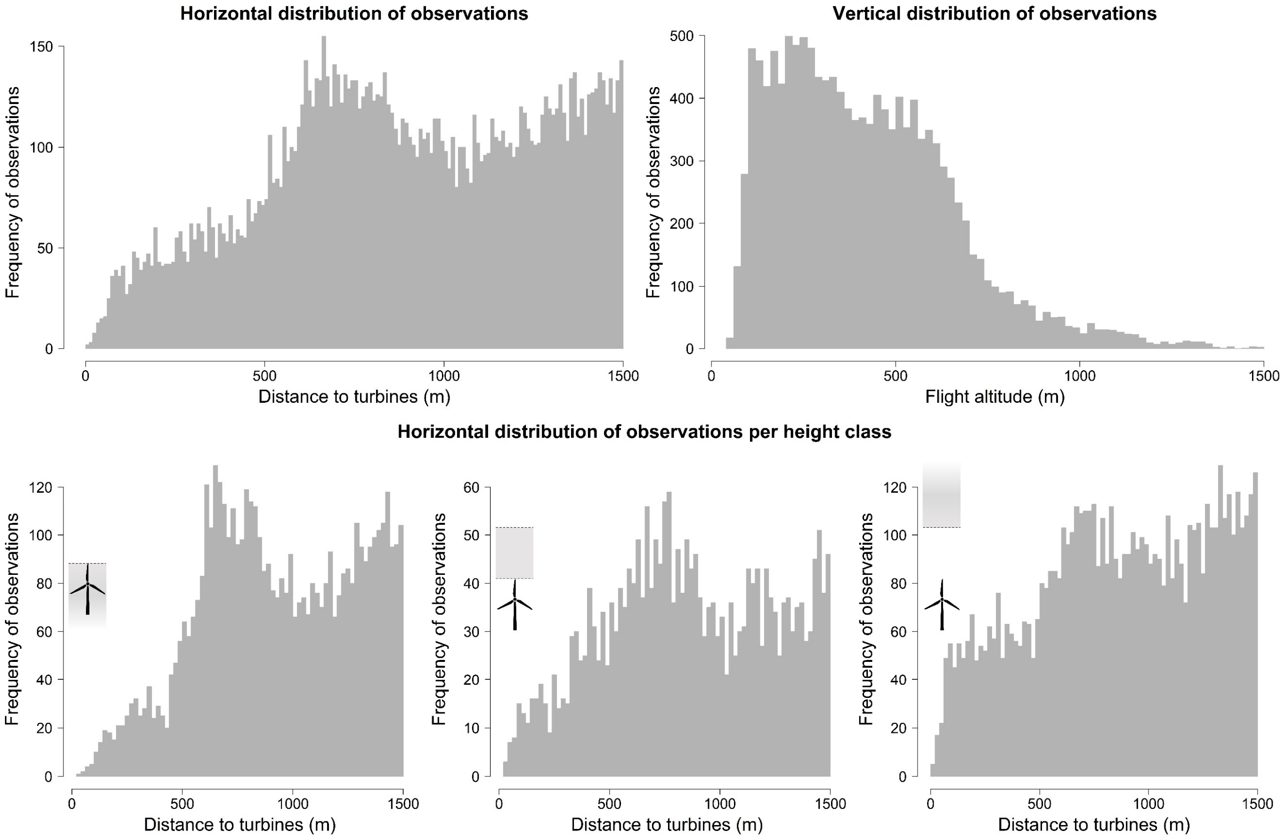

Figure S5. Distribution of observations included in the modelling dataset. Top panels: distribution of observations with the distance to turbines and in altitude. Bottom panels: distribution of observations with the distance to turbines for each flight height class considering the height of the turbines. Top right panel plot omits 37 observations presenting flight altitudes between 1500 and 2582 m.

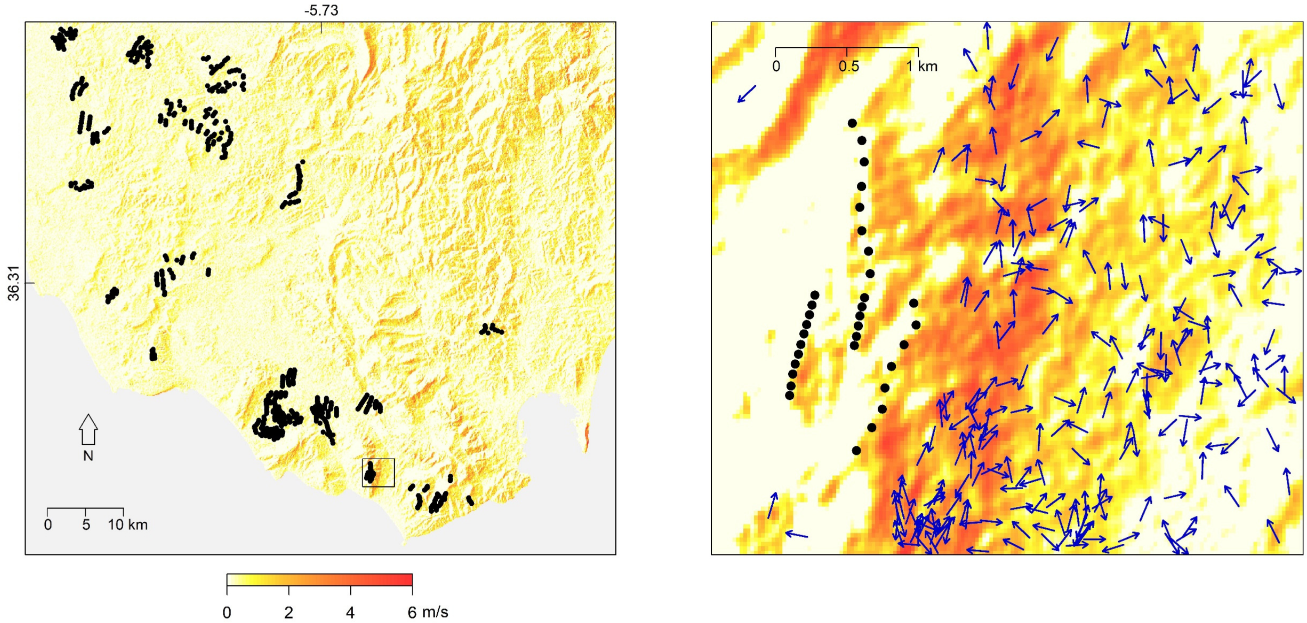

Figure S6. Interaction between bird flight directions (blue arrows in the right panel) and orographic uplift (background map in both panels). We represented only 20% of bird directions recorded in order to allow a clear visualization of main patterns. These observations were selected randomly from the full dataset. Orographic uplift velocity is represented for average wind conditions experienced by the birds during data collection. Terrain aspect and slope used to map uplift velocity were retrieved from a publicly available digital elevation model (https://lpdaac.usgs.gov).

Table S1. Variance inflation factors (VIF) of binomial Generalized Linear Mixed Models (GLMMs) relating the probability of birds to be oriented towards turbines to individual traits and environmental variables. VIF quantifies the severity of multicollinearity in regression models. VIFs of "thermal uplift" and "turbine height" were slightly inflated in the model will all predictors, thus we decided to include these variables in two separate models (see methods for details). OU - Orographic uplift; WCTT - Wind component towards turbines; TU - Thermal uplift; TH - Turbine height.

Model	Age	Sex	OU	WCTT	TU	TH
With all predictors	1.10	1.07	1.31	1.36	2.73	2.52
Without turbine height	1.10	1.07	1.30	1.32	1.11	-
Without thermal uplift	1.07	1.07	1.28	1.29	-	1.03

References

1. Bjornstad, O. N. *ncf: Spatial Covariance Functions*. (R package version 1.2-6, 2018).

2. R Core Team. *R: A language and environment for statistical computing*. (R Foundation for Statistical Computing., 2020).
